# Diversity and Niche of Archaea in Bioremediation

**DOI:** 10.1155/2018/3194108

**Published:** 2018-09-03

**Authors:** Mark James Krzmarzick, David Kyle Taylor, Xiang Fu, Aubrey Lynn McCutchan

**Affiliations:** School of Civil and Environmental Engineering, College of Engineering, Architecture, and Technology, Oklahoma State University, Stillwater, OK 74078, USA

## Abstract

Bioremediation is the use of microorganisms for the degradation or removal of contaminants. Most bioremediation research has focused on processes performed by the domain *Bacteria*; however, *Archaea* are known to play important roles in many situations. In extreme conditions, such as halophilic or acidophilic environments, *Archaea* are well suited for bioremediation. In other conditions, *Archaea* collaboratively work alongside *Bacteria* during biodegradation. In this review, the various roles that *Archaea* have in bioremediation is covered, including halophilic hydrocarbon degradation, acidophilic hydrocarbon degradation, hydrocarbon degradation in nonextreme environments such as soils and oceans, metal remediation, acid mine drainage, and dehalogenation. Research needs are addressed in these areas. Beyond bioremediation, these processes are important for wastewater treatment (particularly industrial wastewater treatment) and help in the understanding of the natural microbial ecology of several *Archaea* genera.

## 1. Introduction

The contamination of soil, sediment, and water from industrial and other human inputs is widespread and poses a threat to human and ecological health. Bioremediation is the use of microbes for the beneficial removal of contaminants of concern [1]. The microbial processes involved in bioremediation are normally natural components of respiration or adaptation, often a component of carbon cycling or metal redox cycling. Thus, bioremediation often occurs without direct intervention; however, biostimulation (the addition of nutrients or adjustment of conditions) and bioaugmentation (the addition of microbes capable of bioremediation) are often important for the complete removal of contaminants within an economical timeframe. The field of bioremediation research has traditionally focused heavily on processes from the domain *Bacteria*, which has a large diversity of bioremediation applications. In many applications where *Bacteria* are the key players in bioremediation, however, *Archaea* are often involved as well. In “extreme” environments, archaeal processes are of particular interest for bioremediation. Many *Archaea* are extremophiles, capable of living in environments considered uninhabitable by most other organisms, and many extreme environments become contaminated and are in need of remediation. Furthermore, many industrial wastewaters have hypersaline, hyperthermal, metallic, and/or an acidic or alkaline pH, where extremophilic *Archaea* have the potential to play key functions for contaminant removal.

This manuscript aims at providing an overview of the various roles that *Archaea* have in bioremediation. This review is meant to be comprehensive but with a particular focus on recent contributions. Both pure culture and mixed community studies are included in the review. The review does not cover nutrient cycling. Nor does it explicitly cover wastewater treatment or provide any explicit review of the environmental microbiology of *Archaea*; however, bioremediation is heavily interconnected to these areas. The review summarizes major findings and suggests future areas of research needed to strengthen our understanding of the contributions of *Archaea* in bioremediation. Though many chapters and reviews exist that encompasses pieces of the topics below, as of the submission of this article, the authors have not uncovered any other comprehensive review that focuses purely on *Archaea* in the bioremediation area.

## 2. *Archaea* in the Degradation of Organics in Hypersaline Environments

Perhaps, the most developed research area that connects *Archaea* to bioremediation lies within the degradation of organics in hypersaline environments. Natural hypersaline environments include salterns, salt lakes, salt marshes, salt flats (sabkhas), and oil and gas production wastewaters. The contamination of these environments with crude oil is common, and about 5% of the chemical, pharmaceutical, and oil industries have highly saline wastewater effluents in need of treatment [2]. Members of both *Bacteria* and *Archaea* are known to inhabit such environments and these are often referred to as “halobacteria” and “haloarchaea,” respectively. Recent reviews have focused on hydrocarbon degradation by halobacteria and haloarchaea [3–5], the biotechnological potential of the hydrolytic enzyme [6], the biodiversity of microbial communities in halophilic environments [7, 8], the potential of haloarchaea in bioremediation processes [9], and the growing rate of research of haloarchaea in bioremediation [10]. Recently, a new database—called HaloDom—has compiled all isolated halophilic species into a single online resource [11]. Many *Bacteria* can degrade at salinities of up to 15% such as strains of the genera *Ralstonia*, *Halomonas*, *Dietzia*, and *Alcanivorax* [12, 13]. Here, an overview of the haloarchaeal strains isolated on the ability to degrade hydrocarbons, such as crude oil, is provided.

**Figure 1:**
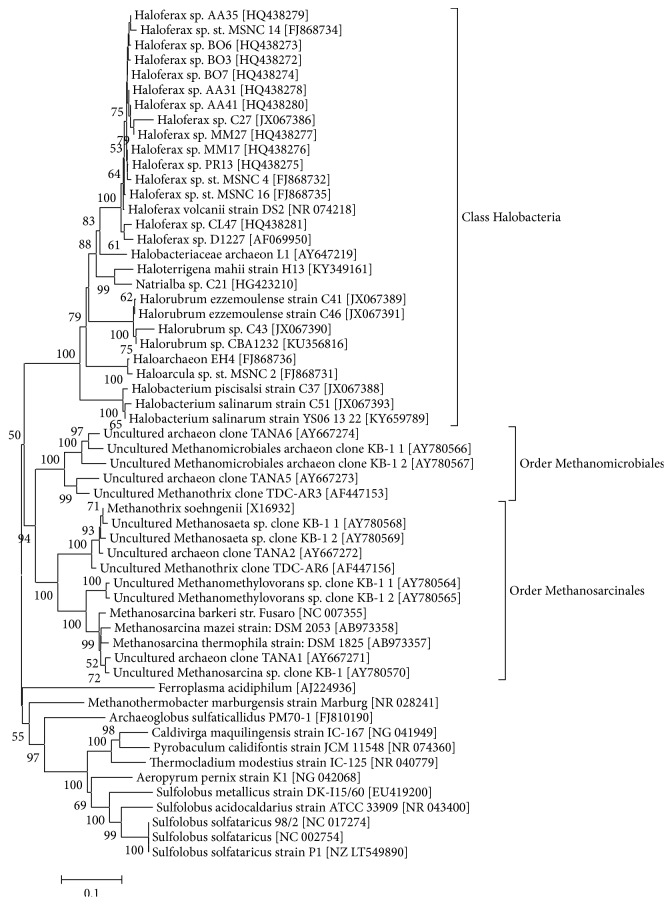
Phylogenetic analysis of strains, or related strains, of the *Archaea* discussed in this manuscript. Alignment and tree analysis was performed in MEGA 6.0 [167]. Sequences were imported from GenBank, alignment was performed with MUSCLE, tree was built with neighbor-joining method with 1000 bootstraps, and evolutionary distances were inferred with maximum composite likelihood method.

The haloarchaea cluster into a single class (the class *Halobacteria*) within the phylum *Euryarchaeota*. They are typically cultured at neutral pH and temperatures of 30-45°C, and they require high salinities of 1.8–5.0 M NaCl [14–17]. Many strains have been traditionally isolated on a standard nutrient media that contains heterotrophic carbon and energy sources [15].  Table 1 lists the strains associated with hydrocarbon degradation and their degradative abilities. Additionally, a phylogenetic tree of many of these strains (where nearly full-length 16S rRNA gene sequences were available), as well as other strains and phylogenetic groups discussed in this manuscript, is shown in Figure 1. The metabolic capabilities of haloarchaea for hydrocarbon degradation appear vast, and these *Archaea* all inhabit a close phylogenetic association.

The connection between the haloarchaea and the degradation of crude oil and xenobiotic pollutants extends past three decades. A haloarchaea strain named EH4, later determined to be closely related to *Haloarcula vallismortis* [18], was isolated in 1990 from a salt marsh in France and found able to degrade various aliphatic and aromatic hydrocarbons [14]. The discovery of hydrocarbon-degrading haloarchaea was independently confirmed with a manuscript published in 1991 reporting the isolation of a *Halobacterium* strain from a hypersaline wastewater in Russia that degrades alkanes [19]. *Haloferax volcanii* strain D1227 was then isolated from a saline oil brine from Michigan (USA) on monoaromatic carboxylic acids as sole carbon and energy sources [20] and later found to degrade 3-phenylpropionate [21]. *Haloferax mediterranei* st. M-11 was isolated from the brine of the Kalamkass oil field (Mangyshlak, Kazakhstan) [22]. *Haloarcula* st. D1 was then isolated and capable of aerobically degrading 4-hydroxybenzoic acid which is a pollutant in certain industrial wastewaters [23]. The degradation pathway consisted of a gentisate-1,2-dioxygenase pathway which was found key in the degradation pathways for *Haloferax volcanii* st. D1227 as well [24, 25]. A sampling of hypersaline lakes in Turkey resulted in 33 isolates of *Halobacteriaceae* across 9 genera [26]. Though these isolates were not directly tested for degradation of crude oil or related hydrocarbons, all 33 isolates tested positive for catalase and oxidase activity and 15 tested positive for Tween 80 hydrolysis [26]. A recent manuscript reported the isolation of four further *Halobacteriaceae* that could also hydrolyze Tween 20 and Tween 80 [27]. Though the Tween 80 and Tween 20 tests are used as a standardized physiological lipase test for microbes [28], it is potentially of particular interest in bioremediation because Tween 80 and related compounds are used as surfactants in oil spill remediation and in hydraulic fracturing mixtures [29, 30].

The study of haloarchaea in bioremediation has gained significant traction in recent years. Four heptadecane-degrading halophilic archaeal strains were isolated from an uncontaminated salt crystallization pond in Camargue, France (*Haloarcula* st. MSNC 2, *Haloferax* st. MSNC 2, *Haloferax* st. MSNC 14, and *Haloferax* st. MSNC 16) [18]. *Haloferax* st. MSNC 14 also grew on phenanthrene while the other three isolates could not [18]. Later research found that *Haloferax* st. MSNC 14 produced surfactants during growth on *n*-heptadecane, pristane, and phenanthrene, but not during growth on acetate [31]. Thus, it was able to increase the bioavailability of low-solubility hydrocarbons during their degradation [31]. Four strains were also isolated from soil and water in a hypersaline coastal area of the Arabian Gulf (*Haloferax* st. HA-1, *Haloferax* st. HA-2, *Halobacterium* st. HA-3, and *Halococcus* st. HA-4) with a multitude of alkane and aromatic degradation abilities [16]. Ten strains of *Haloarchaea* closely related to *Haloferax* were isolated from salt marshes, salterns, crystallizer ponds, salt flats, and the Dead Sea and were found to degrade a mixture of polycyclic aromatic hydrocarbons and crude oil [17]. This study also found that *Haloferax volcanii* st. DS2 could degrade these polycyclic aromatic compounds [32]. This strain, which was isolated from the Dead Sea on glycine and yeast autolysate [33], has just prior had its genome sequenced [34]. *Haloterrigena mahii* sp. H13, collected from a saltern pond in San Diego, CA, USA, also had its genome sequenced and contains genes that may be involved in the degradation of 1,2-dichloroethane, naphthalene/anthracene, *γ*-hexachlorocyclohexane, 1-/2-methylnapthalene, and benzoate [17, 35]. A literature search has not uncovered any research that directly tested the aforementioned biodegradation capabilities with this pure culture.

The diversity of haloarchaea-degrading hydrocarbons, and of xenobiotics that they can degrade, has been expanding. A strain of *Halobacteriaceae* (named L1) was isolated from the Dead Sea and could grow on benzoic acid [36]. *Natrialba* sp. st. C21 has also been isolated from oil-contaminated saline water in Ain Salah, Algeria [37]. This strain can degrade phenol, naphthalene, and pyrene through an ortho-cleavage pathway and exhibits catalase, oxidase, and Tween 80 esterase activity [37]. Acikgoz and Ozcan [38] found eight *Halobacteriaceae* out of a screening library of 103 isolates that could degrade and tolerate above 200 ppm phenol. The fastest phenol-degrading strain was identified as a *Haloarcula* sp., but more detailed phylogenetic characterization was not provided [38]. In another study, nine isolates were found that can use aromatic hydrocarbons for carbon and energy sources [39]. These isolates were identified as members of *Haloferax* sp. (isolates C-24 and C-27), *Halobacterium piscisalsi* (st. C-37), *Halobacterium salinarum* (st. C-51), *Halorubrum ezzemoulense* (st. C-41 and C-46), and *Halorubrum* sp. (st. C-43), and two strains (C-50 and C-52) reported with less than 93% 16S rRNA gene identity to any isolated strains [39]. Upon inspection of the deposited sequences in NCBI's GenBank, the sequence for strain C-50 appears to have poor sequence quality; a BLAST search of the first 280 bp recovered zero alignments to sequences in GenBank. Strain C-52 has 99% identity along the more recently deposited 16S rRNA gene of *Halorubrum trapanicum* CBA1232, which has a deposited genome (NCBI BioProject PRJDB4921); however, no publications are associated with this genome [40]. All nine strains degraded naphthalene, phenanthrene, and pyrene, and all but strain C-37 and C-51 degraded p-hydroxybenzoate [39]. Degradation in all cases was through ortho-oxidation through a catechol 1,2-dioxygenase or a protocatechuate 3,4-dioxygenase pathway [39]. A microbial community enriched from the Great Salt Lake (Utah, USA) consisted of several genera entirely of the class *Halobacteria*, with 91% belonging to the genera *Halopenitus* as determined by 454 sequencing of 16S rRNA genes [41]. This community could grow on 4-hydroxybenzoate but not the other carbon sources tested, and the degradative pathways and genes were analyzed with PCR approaches of functional genes [41].

Though the isolation of haloarchaeal strains from contaminated sites is successful and haloarchaea are often found in natural environments (i.e., [42, 43]), the understanding of the microbial ecology of these strains on oil contamination under in situ conditions is not well developed. A few studies investigating the distribution of the haloarchaea have been done. The archaeal community in a saline-alkali soil in the Dagang Oilfield (China) differed significantly along a petroleum contamination gradient, with four groups of *Archaea*, including *Haloferax* and *Natronomonas*, being abundant in the contaminated soils while five different groups of *Archaea* were dominant in noncontaminated soils [44]. Other studies have profiled further diversity of haloarchaeal groups in oilfield sites, including the genera *Halalkalicoccus*, *Natronomonas*, *Haloterrigena*, and *Natrinema*, suggesting that varied haloarchaea are widely present in these contaminated environments [45]. Though *Haloferax* has a number of isolates known to degrade aromatics, *Natronomonas* is not as well established to oil degradation, though it does contain fatty acid degradation pathways and is thus putatively able to degrade alkanes [46]. Thus, these genera are likely degrading the organics in situ. In contrast, in a hypersaline-produced water from the Campos Basin (Brazil) contaminated with phenol and aromatics, the archaeal community consisted of no detected haloarchaea in situ but was rather dominated by methanogens (59% *Methanosaeta* and 37% *Methanoplanus*) [47]. Methanogens have a role in the final degradation of hydrocarbons in coculture with hydrocarbon-degrading *Bacteria* (see below); the presence of methanogens and the lack of haloarchaea suggest a highly reduced environment. Hydrocarbon-degrading halophilic bacteria (specifically, *Halomonas*) were isolated from these waters and could degrade these contaminants, especially with biostimulation [48]. The contaminants in this production water were also degraded more significantly in a previous study with the bioaugmentation of haloarchaea strains [32]. The bacteria *Halomonas* and haloarchaea survive in similar salinities and contain similar degradative capabilities [4]; however, it is not known what drives the competitive advantage of one over the other.

Recently, further studies have progressed towards evaluating bioremediation techniques with haloarchaeal communities. A recent study focused on how vitamin amendments may stimulate crude oil degradation [49]. Vitamin B_12_ enhanced the degradation of crude oil from five *Archaea* strains tested (*Haloferax lucentense* st. AO1, *Halobacterium salinarum* st. AO2, *Halobacterium piscisalsi* st. AO3, *Haloferax mucosum* st. AO4, and *Halobacterium sulfurifontis* st. AO5) [49]. Pyridoxine enhanced the biodegradation of oil by four of these strains (A01, A02, A04, and A05), riboflavin enhanced the degradation by three strains (A01, A02, and A05), folic acid enhanced the degradation by three strains (A01, A03, and A05), and thiamin enhanced the degradation by one strain (AO5), but biotin did not enhance oil degradation significantly by any of the five strains [49]. The biostimulation with vitamins is not surprising, as earlier work has shown that a nutritional yeast extract amendment significantly increases hydrocarbon degradation [32]. The strains were found to also degrade Tween 80, *n*-octadecane, and phenanthrene and were also enhanced with 0.75 M KCl and 2.25 M MgSO_4_ [49, 50]. In another study, continuous illumination and casamino acids were found to increase oil biodegradation by mixed cultures dominated by *Haloferax* sp. and by four isolates (two identified as *Haloferax*, one as a *Halobacterium*, and one as a *Halococcus*) [51]. *Haloferax elongans* st. M4 and *Halobacterium salinarum* st. M5 were found capable of being cultured onto a *Bacteria*-*Archaea* biofilm community for the degradation of crude oil, *n*-hexadecane, and phenanthrene [52]. Such biofilm communities have advantages in bioremediation technologies. There too, vitamins stimulated crude oil degradation in the biofilm [52]. In yet another study with a mixed community of *Bacteria* and *Archaea*, the addition of casamino acids and citrate was required for oil degradation and the microbial community dynamics were observed [53]. After adding crude oil to the culture, biotic degradation could not occur and the archaeal community shifted away from what was previously high levels of *Haloquandratum*, to one in which only *Natronomonas* spp. remained, while the bacterium *Salinibacter* was selected [53]. With the additional amendment of casamino acids and citrate, the community could degrade oil with an archaeal enrichment of *Haloarcula*, *Haloterrigena*, and *Halorhabdus* [53]. A recent study investigated the biostimulation of oil-degrading cultures derived from a hypersaline sabkha and found that Fe^+3^, Ca^+2^, Mg^+2^, K^+^, animal blood, and commercial yeast all had a stimulatory effect towards oil degradation [54]. Haloarchaeal communities were dominated by *Haloferax* spp. and *Halobacterium* spp., and eight strains were isolated (two associated with *Halobacterium noricense*, two with *Haloferax larsenii*, a *Halobacterium salinarium*, and a *Halobacterium* sp.) [54]. These strains could grow on a variety of alkanes and aromatics and degraded between 22 and 36% of amended crude oil over 2 weeks [54].

Cocontamination of different types of pollutants often complicates bioremediation, and a recent study has investigated the effect of heavy metal cocontamination with hydrocarbon degradation in hypersaline systems [55]. Strains of both *Archaea* (a strain of *Haloferax elongans* and a *Halobacterium salinarum*) and *Bacteria* (a strain each of *Arhodomonas*, *Marinobacter*, and *Halomonas*) were inhibited with elevated levels of Hg, Pb, Cu, Cd, and As and were more sensitive to these metals in the presence of crude oil [55]. Overall, the archaeal strains had less tolerance for heavy metals than three halophilic/halotolerant *Bacteria* tested, though the bacterial genus *Kocuria* had similar levels of sensitivity to heavy metal toxicity [55]. For the *Haloferax elongans*, Fe^III^ amendment lessened the toxicity of Hg, Pb, Cu, and Cd, while for the *Halobacterium salinarum*, Fe^III^ amendment lessened the toxicity of Cu, Cd, and As and proline lessened the toxicity limit of Cd [55]. For the *Halobacterium salinarum*, the rate of crude oil consumption was tested under heavy metal stress with and without Fe^III^ or proline amendment. The crude oil degradation rate increased significantly under Hg or Pb stresses with Fe^III^ or proline amendment, while the enhancement of oil consumption rates in Cu-, Cd-, and As-stressed cultures were more nuanced [55]. At low-salt concentrations (<1.5 M), many of these heavy metals, to a certain concentration, increased cell growth presumably from affecting cytoplasmic osmolality [55]. In previous research, the strain *Haloferax* sp. st. BBK2 was affected by 0.5 mM concentrations of Cd but was resistant to Cd toxicity up to 4 mM levels and it accumulated Cd intracellularly [56].

The progress within this area from simple discovery to in-depth biostimulation analysis over the last decade is tremendous despite the relatively few investigators that have been steadily producing significant findings in this area. The diversity of strains and isolates within the haloarchaea is large, but not exhaustive [41, 57]. The study of haloarchaea benefits from moderate growth rates (doubling times of ~24–32 hr), fruitful isolation attempts, and easy culturing conditions (aerobic, diverse organic substrates, etc.) [14–17]; however, more molecular-based research to monitor and detect in situ degradation is needed to better understand these archaeal biodegradation processes in contaminated hypersaline environments. Though they have relatively warm temperature preferences (generally greater than 30°C) and have vitamin needs [14–17, 32, 49], the broad distribution of haloarchaea in hypersaline environments, the broad metabolic capabilities found on xenobiotics and crude oil, and the relatively quick degradation rates all provide promise that if properly stimulated, bioremediation of hydrocarbons in hypersaline environments should proceed quickly.

## 3. Degradation of Organics with Thermophilic *Sulfolobus solfataricus*

A few strains of thermophilic and acidophilic *Archaea* have been found capable of pollutant degradation. Such biodegradation capabilities are of interest, as many industrial wastewater streams are hot. Genomic sequencing of *Sulfolobus solfataricus* st. P2 found genes for aromatic degradation and it was found to be able to degrade phenol aerobically through *meta*-ring cleavage [58]. A strain of the closely related thermophilic *Sulfolobus solfataricus* st. 98/2 was later found to be able to degrade phenol at 80°C and 3.2 pH [59, 60] through *meta*-ring cleavage also [61]. A dienelactone hydrolase from *Sulfolobus solfataricus* st. P1 was also identified and characterized [62]. This enzyme is important for chloroaromatic degradation, such as 2,4-dichlorophenoxyacetic acid [63], though direct testing of this enzyme on chloroaromatics was not reported. To our findings, this seems to be the extent of current research on *Sulfolobus* in terms of bioremediation applications, but a review of *Sulfolobus* in broader biotechnology applications has recently been published [64]. This research field is still developing and there are likely more thermophilic hydrocarbon degraders; however, culturing thermophilic strains is difficult due to maintaining high temperatures for cellular growth, the increased volatility of the hydrocarbons at high temperatures, and for aerobes, the low oxygen solubility at high temperatures.

## 4. Degradation of Hydrocarbons in Soils with *Archaea*

In nonextreme environments, *Bacteria* are better known to perform the degradation of hydrocarbons; however, *Archaea*, particularly the methanogens, are often a component of the degradation process. Hydrogenotrophic and acetoclastic methanogens convert hydrogen and acetate, respectively, to methane gas in anaerobic conditions [65]. In degradative processes where hydrogen or acetate are waste products, these methanogens can thus increase the thermodynamic favorability by reducing hydrogen and acetate concentrations and in effect drive the degradative process forward [66]. This forms a syntrophic relationship between *Bacteria* that degrades the compound of interest and the methanogenic *Archaea* that removes the waste products of that degradation [67]. Acetoclastic methanogens are found in the order *Methanosarcinales*, notably the genera *Methanosaeta* and *Methanosarcina*, while hydrogenotrophic methanogens are found in the orders *Methanococcales*, *Methanobacteriales*, *Methanosarcinales*, *Methanomicrobiales*, *Methanopyrales*, and *Methanocellales* [68]. Here, we review the key roles of *Archaea* in soils and freshwater systems contaminated with hydrocarbons. A recent review was published that more broadly covers microbial community responses to petroleum contamination [69].

Two decades ago, an analysis of the microbial communities in a jet fuel and chlorinated solvent-contaminated aquifer found that *Methanosaeta* spp. dominated the archaeal community and it was proposed that it performs the terminal step in hydrocarbon degradation in methanogenic zones [70]. Soon thereafter, enrichment cultures showed that long-chain alkanes can be degraded anaerobically to methane with a culture of *Syntrophus* spp. (including one closely related to a sequence recovered from the jet fuel/chlorinated solvent-contaminated aquifer in [69]) and both acetoclastic (*Methanosaeta* sp.) and hydrogenotrophic (*Methanoculleus* sp. and *Methanospirillum* sp.) methanogens [71]. Since then, many field studies with in situ hydrocarbon degradation have investigated for the presence of methanogenic *Archaea*. Soil contaminated with petroleum and undergoing remediation was found enriched significantly for *Methanosarcinales* strains with a denaturing gradient gel electrophoresis (DGGE) method [72]. *Methanomicrobiales*, *Methanosarcinales*, *Methanobacteriales*, and *Thermoplasmatales* were all found in other soil samples contaminated with petroleum hydrocarbons [73]. High abundances of *Methanosaeta* were observed in a diesel-contaminated soil—up to 30% of all 16S rRNA genes in some of the samples [74]. This compares to normal abundances of 2% *Archaea* in natural soils, which are also typically dominated by *Crenarchaeota* and not the *Euryarchaeota* of which the methanogens belong [75]. Processed oil sands were also found to contain archaeal communities dominated by the acetoclastic *Methanosaeta* spp. [76]. A coculture of *Anaerolineae* and *Methanosaeta* was found to predominate in an alkane degradation culture over 1300 days with similar 16S rRNA gene concentrations of each, presumably with *Anaerolineae* breaking down alkane chains through acetate and *Methanosaeta* fermenting acetate into methane [77]. Another study found that the genus *Methanoculleus* was the more abundant methanogen in an anaerobic alkane degrading culture containing the bacteria *Thermodesulfovibrio* and *Anaerolineaceae* [78].

Often, the diversity of *Archaea* detected in hydrocarbon degrading cultures is low but the diversity of *Archaea* in one heavy crude oil-contaminated soil was found to be higher than the diversity of *Archaea* in a pristine soil [79]. Clone libraries indicated that the contaminated soil contained many members of deeply branching *Methanomicrobiales*, *Halobacteriales*, *Methanosarcinales*, and many *Euryarchaeota* and *Crenarchaeota* of uncultured genera, while the pristine soil only contained *Natronomonas*-like sequences among the *Archaea* [79]. In a hydrocarbon-contaminated sludge from an oil storage facility, *β-Proteobacteria* was found in coculture with a diverse archaeal community consisting of *Thermoprotei* (54%), *Methanocellales* (33%), and then *Methanosarcinales/Methanosaetacaea* (8%) [80].

The study of syntrophic hydrocarbon degradation has advanced to studying systems under biostimulation conditions. The anaerobic degradation of benzene is oftentimes slow or nonexistent [4]. In a field-based study comparing the natural attenuation of B20 biodiesel blend and a biostimulation with an ammonium acetate injection, it was found that *Archaea* populations significantly increased from less than 10^3^ to 3.7 × 10^8^ 16S rRNA genes·g^−1^ under the biostimulation conditions commensurately with enhanced BTEX degradation [81]. Conversely, in a recent study of an Alpine Petroleum-contaminated site, the archaeal community was mostly found unchanged on the phyla level (based on read depth analysis of a 16S rRNA gene amplification) and overall archaeal abundance (measured with qPCR) decreased during fertilization biostimulation or increased temperature [82]. The only archaeal enrichment appeared to be *Woesearchaeota* which became more abundant compared to other archaeal phyla with a temperature increase to 20°C [82]. This study did not report data on finer phylogenetic scales.

The syntrophic relationship between hydrocarbon-degrading *Bacteria* and methanogenic *Archaea* is not always present in degradation cultures. *Euryarchaeota* and *Thaumarchaeota* completely disappeared in one set of microcosms amended with spent motor oil [83]. Similarly, Illumina sequencing of a 16S rRNA gene amplification did not widely detect *Archaea* in one petroleum enrichment culture [84]. A GeoChip analysis of the archaeal community in a different study found that archaeal abundance was negatively impacted by oil contamination in an aerobic soil with numbers reduced to 10% of the archaeal abundance in noncontaminated soil [85]. A DGGE-based community profile of an Antarctic soil contaminated with diesel under various remediation conditions found no substantial differences in the archaeal community during bioremediation [86]. Another study found that *Archaea* were scarce (<1% of the population) in an aquifer above a coal-tar DNAPL with only a low abundance of methanogens [87]. Other than the reduced redox conditions required for methanogenesis, it is not clear why *Archaea* respond strongly to oil contamination in certain environments and not others.

A diverse and varying dominance of archaeal members (as well as bacterial members) exists in soils and groundwater during hydrocarbon bioremediation. Controlled experiments in which physicochemical conditions (such as redox, salinity, temperature, and trace element availability) are varied in hydrocarbon-contaminated soils may help determine the role that these factors play in selecting the specific archaeal communities (if any at all) that are stimulated. The research in this area also uses a variety of methodologies to study the *Archaea*, and similar methodologies (clone libraries) still often use different primer sets. Studies in which these methodologies are compared for the same sample would help elucidate the extent that the varying results above are a function of the chosen methodology.

## 5. *Archaea* in the Degradation of Oil in Oceans and Marine Sediments

The role of *Archaea* in the degradation of oil in marine systems is oftentimes unclear as well. It is believed that *Bacteria* play the dominant role in oil biodegradation in oceans [88], but the role of *Archaea* in oil degradation in oceans is not fully understood. *Archaea* in many studies have been found to be sensitive to oil compounds. In a lab-based study of beach sediment microcosms, *Archaea* 16S rRNA genes became difficult to amplify with a PCR method after incubation with oil, suggesting a large decrease in archaeal populations [89]. That study however only detected two tight clusters of *Archaea* in its analysis, a group of Marine Group II *Euryarchaeota* and a group of *Crenarchaeota* [89]. A later study found that the nitrifying *Nitrosopumilus maritimus*, a member of the Marine Group I *Archaea*, was also very sensitive to crude oil presence [90]. In another study, the oil degrading bacteria that were found to grow were heavily dependent on temperature but the archaeal community structure was minimally affected [91]. The study also observed few *Archaea* groups—predominately a tight phylogenetic group of Marine Group II *Archaea* and eight other OTUs related to *Euryarchaeota* and *Thaumarchaeota* [91]. The isolation of hydrocarbon-degrading strains in coastal sediment contaminated with petroleum off of the coast of Sicily (Italy) recovered only isolates from the domain *Bacteria* [92]. The natural diversity of archaeal communities were determined with DGGE and was found to consist of uncultured *Crenarchaeota* and *Thaumarchaeota* which did not significantly change in crude oil-amended microcosms [92]. Though members of *Thaumarchaeota* are hypothesized to be able to aerobically degrade crude oil [93], no direct evidence with cultured strains yet exists.

Other studies have detected shifts in archaeal communities that suggest that some *Archaea* may at times play a role in degradation. One study tested the change in the archaeal community before and after adding either heptadecane, naphthalene, or crude oil in seawater and marine sediment at two locations near Rio de Janeiro (Brazil) [94]. While no *Archaea* could be identified in the water samples, the archaeal community in the marine sediment uniquely changed for each of the hydrocarbons that were added [94]. The method detected primarily uncultured *Archaea*, which were mostly *Euryarchaeota* [94]. In a field study, a DGGE analysis of archaeal 16S rRNA genes indicated that oil contamination in mangrove sediments differed compared to a pristine site [95]; again, the method predominately detected uncultured groups of *Archaea*. In a recent survey of Atlantic and Mediterranean coastal sediments around Europe, the presence and abundance of the Miscellaneous Crenarchaeotic Group (MCG) were also found to correlate to oil-contaminated sediments [96]. These findings suggest that some uncultured groups of *Archaea* may have roles in oil degradation in marine systems.

Methanogens have been connected to hydrocarbon degradation in some marine systems as well. Methanogenesis increased commensurately with hydrocarbon degradation in microcosms seeded with contaminated sediments taken from Halic Bay (Turkey) and stimulated with phosphorus and/or nitrogen [97]. A research study also found that adding methanol or acetate could stimulate degradation of petroleum hydrocarbons in marine sediment [98]. The acetoclastic methanogenic *Methanosarcinales* increased in the sediment with acetate stimulation and temporarily with methanol stimulation [98]. *Methanomicrobiales*, which are hydrogenotrophic methanogens, increased with methanol stimulation as well, but not with acetate stimulation [98].

Though haloarchaea contain many strains that require high levels of NaCl, recent evidence suggests that marine systems have phylogenetically related strains as well. Samples taken from the Amazon equatorial ocean basin and amended with oil droplets had significant variation in the community composition of the *Archaea* domain upon oil biodegradation as detected with metagenomic techniques, including a relative enrichment of the *Halobacteriaceae* [99]. In a mesocosm study of archaeal and bacterial diversity from oil contamination in mangrove sediments, bacterial diversity was more significantly affected from oil contamination than archaeal diversity [100]. The genus *Nitrosopumilus*, common in marine systems, was inhibited with oil degradation, but the read depth for the family *Halobacteriaceae* was stimulated from combined oil and nitrate additions, of which members related to *Haloferax* increased marginally with oil additions [100]. *Archaea* was not found to be affected by oil contamination in the coastal water of the Gulf of Finland, but they were impacted in the coastal sediments [101]. The *Halobacteriaceae* was significantly more abundant where the sediment was contaminated with oil [101]. Archaeal cytochrome 450 and retinol metabolism pathways were enhanced where oil was also present which signifies active oil degradation [101]. Altogether, these results indicate that some haloarchaea likely have roles in oil biodegradation at least in sediments. Degradation of oils in sediments is important, as coastal systems are oftentimes more contaminated with oil than open oceans.

In many of the studies above, a limited diversity of *Archaea* was measured, typically with methods relying on a PCR amplification with universal primers followed by an analysis. Interpreting results from these studies should be done cautiously because amplification-dependent methodologies may miss clades of *Archaea* due to primer mismatching and/or PCR biases [102]. With modern metagenomic sequences, it may be worthwhile to reexamine old assumptions based on these results. Indeed, recent metagenomic-based methods are elucidating much greater diversity of *Archaea* in marine systems than the earlier studies using methods dependent on PCR amplification were detecting (i.e., [99]).

## 6. Archaea in Heavy Metal Remediation

Bioremediation of metals can take many forms [103]. Oftentimes, it involves the redox cycling of the metals for the conversion of toxic redox states to nontoxic redox states. Alternatively, redox cycling may convert soluble metal redox states to insoluble redox states, or vice versa, and the effect of which is precipitation or mobilization of the metal. Additionally, metals may be removed through reactions that permit volatilization of heavy metals or through sorption into biomass. These processes are also important for radioactive metals [104], but *Archaea* are poorly studied in this area despite some archaeal strains having high tolerance of radioactivity [105]. A recent review over the bioremediation of heavy metals was published, but did not address *Archaea* [106]. A comprehensive review of metal-tolerant thermophiles has been published recently including significant information regarding *Archaea* and the significant context in terms of bioremediation [107]; thus, here, we do not cover thermophiles and metal bioremediation in as much detail.

Arsenite (As^III^) is a toxic form of arsenic, but it can be oxidized to less toxic arsenate (As^V^). In a study of an acidic, sulfuric thermal spring in Yellowstone National Park (USA), arsenite oxidation coincided with the appearance of unisolated *Crenarchaeota* and *Euryarchaeota* and it was thus hypothesized that *Archaea* could oxidize arsenite [108]. In earlier work, the *Sulfolobus acidocaldarius* st. BC was indeed confirmed to oxidize arsenite to arsenate [109]. From reviews of the deposited genomic sequences in GenBank, the *Archaea* strains *Aeropyrum pernix* st. K1, *Pyrobaculum calidifontis* st. JCM 11548, and *Sulfolobus tokodaii* st. 7 are found to contain arsenite oxidase genes [110, 111]. A recent metagenomic study of Diamante Lake (Argentina) found a large abundance of arsenate reduction and arsenite oxidation genes and haloarchaea [112]. Fourteen isolates of the genus *Halorubrum* were found to contain arsenite oxidation genes and one strain was confirmed capable of arsenite oxidation [112]. Arsenate reduction by *Archaea* is also common which in turn would increase arsenic toxicity (i.e., [113]).

Mercuric mercury (Hg^II^) is highly toxic and one method of removal is via biological reduction to volatile zero-valent mercury (Hg^0^). This is carried about by enzymes encoded by mercury reductase genes which have been identified in several diverse *Crenarchaeota* and *Euryarchaeota* [114]. A study of a mercury-containing hot spring in Yellowstone National Park (USA) found novel and deeply rooted mercury reductase genes associated with *Archaea* [115]. Mercury reductase was found upregulated in *Sulfolobus solfataricus* and was needed for mercury resistance [116], and mercury volatilization was also measured from *Halococcus*, *Halobacterium*, and, to a lesser extent, *Haloferax* [117]. Direct study of zero-valent mercury volatilization from *Archaea* is otherwise rather scarce. Conversely, mercury methylation by methanogens, which increases toxicity, is well documented [118].

The precipitation of uranium by the reduction of U^VI^ to U^IV^ is one mechanism for the immobilization of uranium in environments where it may impact ground and surface waters [119]. *Pyrobaculum* sp., which are hyperthermophiles, are capable of uranium reduction [120]. These *Archaea* have large redox capabilities for other metals (i.e., [121]) and thus may be beneficial in many types of metal-contaminated hyperthermic waste streams.

Another way in which metals may be bioremediated is via intracellular or extracellular binding or sorption. *Methanobacterium bryantii* was found to excrete extracellular proteins to chelate copper [122]. *Sulfolobus acidocaldarius* was found to bind U^VI^ into organophosphate groups [123]. Halophilic microbes are often able to absorb heavy metals, as well [124]. *Halobacterium* sp. GUSF was found to be able to absorb manganese at high rates and high concentrations [125]. *Halobacterium noricense* was found to adsorb cadmium [126]. As noted above, *Haloferax* st. BBK2 was found to accumulate cadmium intracellularly [56]. The archaeon *Halobacterium noricense* DSM15987 was found to accumulate U^IV^ with phosphoryl and carboxylate groups compared to a direct biosorption process with the bacterium *Brachybacterium* sp. G1 [127, 128]. These results show promise that the haloarchaea can be used in the treatment of hypersaline environments and wastewaters for heavy metal removals.

## 7. *Archaea* in Acid Mine Drainage

Acid mine drainage is a major contributor to water pollution by introducing a highly acidic effluent with toxic metals in solution. Acid mine drainage occurs when oxygen, introduced due to mining activities, reacts with metal sulfide minerals (such as FeS_2_) resulting in the production of sulfuric acid and lower pH; this reaction is often aided by aerobic iron- and sulfur-oxidizing microbes [129]. Many microorganisms including many *Archaea* tolerate and thrive in the acidic and metal dense environments found in acid mine drainage. *Ferroplasma* spp. are acidophilic metal oxidizers with preferences of very low pH (<1.5) and are major players in the production of acid mine drainage and the biogeochemical cycling of sulfur [130, 131]. At Iron Mountain (CA) which has acid mine drainage, *Archaea* are the major proportion of the prokaryotes and *Ferroplasma* dominates (85% of *Archaea*) [130]. Many other *Archaea* are involved in similar ways. For example, *Sulfolobusmetallicus*, which is also acidophilic, thermophilic, and chemolithoautotrophic, can oxidize elemental sulfur and sulfidic ores, producing sulfuric acid and causing the leaching of uranium, zinc, and copper [132]. Exploiting these *Archaea* may be important for mining of metals and biocatalysis under extreme conditions (i.e., [133]) but may not be helpful in an acid mine bioremediation context where increased toxic metal mobility and acidification is typically not a favorable outcome. However, the diversity of the *Archaea* in the order *Thermoplasmatales* and their resistance to toxic metal resistance [134] may prove useful for other metal remediation purposes.

The biological treatment applying sulfate-reducing bacteria is an attractive option to treat acid mine drainage and to recover metals [135]. The process produces alkalinity, neutralizing the acid mine drainage simultaneously. There are two lineages of archaeal sulfate reducers: the *Archaeoglobus*, within the *Euryarchaeota*, and *Thermocladium* and *Caldivirga* within the *Crenarchaeota* [136]. *Archaeoglobus* are thermophilic but not acidophilic [137]. *Thermocladium* and *Caldivirga* are moderately acidophilic and can tolerate pH down to about 2.3 but are still thermophilic and thus are not suitable for acid mine drainage [138, 139].

## 8. Archaea in Reductive Dehalogenation

Reductive dehalogenation removes halides from organic compounds resulting in lower halogenated or nonhalogenated products and is important in bioremediation. This field has been largely focused on the organohalide-respiring *Bacteria* that can use organohalides as terminal electron acceptors. However, the ability of methanogens to dehalogenate has been long established. Many papers were published in the 1980s and 1990s discovering the various substrates subject to dechlorination by methanogens. Various strains of *Methanosarcina* were found to dehalogenate pentachlorophenol [140], perchloroethylene [141], trichloroethene [142], chloroform [143], and trichlorofluoromethane [144]. *Methanobacterium ivanovii* strain T1N was able to degrade pentachlorophenol [140]. Cell suspensions of *Methanosarcina barkeri* (DSM 2948), *Methanosarcina mazei* (DSM 2053) (which was incorrectly referred to as *Methanococcus mazei* despite reclassification 8 years prior [145]), *Methanobacterium thermoautotrophicum* st. Marburg (DSM 2133) (which has since been reclassified as *Methanothermobacter marburgensis* [146]), and *Methanothrix soehngenii* (DSM 2139) dechlorinate 1,2-dichloroethane through dihaloelimination to the product ethylene and through hydrogenolysis to chloroethane [147]. The ability to dehalogenate is likely from the high concentrations of corrinoids, such as cobalamin, in methanogens which are needed for methanogenesis [148, 149]. Corrinoids are able to dehalogenate organics abiotically [150, 151].

*Archaea* are also commonly reported as a part of microbial communities dechlorinating chloroethenes (Table 2). *Methanobacterium congolense* was found in the well-studied chloroethene-dechlorinating ANAS culture [152]. Inhibition of the methanogens with 2-bromoethanesulphonate (BES) was reported to not affect the “ability to dechlorinate trichloroethene completely”; however, further information was not provided [152]. *Methanothrix*, *Methanomethylovorans*, and an unclassified *Archaea* were present in a column treating perchloroethene [153]. At a site undergoing remediation from trichloroethene to ethene, *Methanosaeta* sp., *Methanospirillum* sp., *Methanosarcina*, and an unclassified *Methanomicrobiales* were found present [154]. *Methanosarcina*, *Methanomethylovorans*, *Methanomicrobiales*, and *Methanosaeta* were reported as significant components of the well-studied and highly enriched KB-1 organochloride-dechlorinating culture [155]. *Methanosarcina* was found to be important for the dechlorination of vinyl chloride in an enriched *Dehalococcoides*-containing culture, while *Methanosaeta* had no impact [156]. It was hypothesized that the *Methanosarcina* were producing H_2_ from acetate oxidation for the *Dehalococcoides* in these cultures [156]. Hydrogenotrophic methanogens in other cultures are conversely likely competing for H_2_ substrate with the organohalide-respiring bacteria [157, 158]. Many dechlorinators, such as the versatile *Dehalococcoides*, lack the ability to synthesize needed corrinoids for reductive dehalogenation and instead have genes for corrinoid scavenging and import [159, 160]. Methanogens in these cultures may provide these key corrinoids for the organohalide-respiring bacteria in these communities, though this role may be fulfilled by other corrinoid-producing bacteria [158]. A recent review on cobalamin synthesis in the context of dehalogenation has been published [161]. The ability of methanogens to dechlorinate suggests that these *Archaea* may contribute to dechlorination activities even in systems dominated by organohalide respirers. The roles and antagonism of *Archaea* in reductive dechlorination systems are likely complex. Recent research has started investigating the natural cycling of organohalides but has only thus far focused on *Bacteria* [162–164].

## 9. Research Needs

A primary hurdle in the study of *Archaea* in bioremediation systems is methodological. Many studies on bioremediation do not study archaeal community members explicitly nor have methods that would allow for the discovery of archaeal diversity or activities. Additionally, many methodologies that have been used to study *Archaea* are prone to biases, which may cloud our understanding. A varied number of archaeal and “universal” amplification primer pairs are known and are used to study archaeal diversity [32, 37, 82, 83, 86]. Interpreting results from these methods should be done carefully. PCR amplifications of entire prokaryotes or entire domains are prone to biases, which can underrepresent and overrepresent various microbial community members [102]. Analyses that are based on a high phylogenetic level (i.e., phylum-based analyses) can also hide trends on the finer phylogenetic levels (i.e., genus). Recent publications above often rely on “relative read depth” analysis of the high throughput sequencing of a 16S rRNA gene amplification product to provide quantitative measurement of specific *Archaea* taxonomic groups; however, these methods are still exposed to PCR biases. For analysis of mixed cultures, metagenomic sequencing of unamplified DNA and more quantitative PCR (qPCR) methods should also be used. QPCR has a high sensitivity, can be designed for high specificity, and can be quality controlled [165] and thus makes a superior quantitative method to “relative read depth” analysis which lacks these characteristics. In a recent publication, read depth analysis from an Illumina-sequenced PCR product was able to identify enriched taxonomic groups, but the read depth analysis agreed poorly with the actual quantification with qPCR [164]. Some qPCR methods have been developed for certain *Archaea* (i.e., [155]); however, more methods need to be developed to further extend the study of *Archaea* in mixed microbial communities.

An additional hurdle in studying *Archaea* in bioremediation again is methodological. Dose growth response analysis is often used to measure community members that outcompete others at a given physicochemical condition on a given substrate. One hypothesis of *Archaea* evolution suggests that *Archaea*'s niche and advantage in the environment is operating under energy stress, and thus, dose growth response methods provide conditions where *Archaea* may easily be outcompeted [166]. In the environment, biodegradation activity often occurs in heterogeneous environments with microniches, energy stresses, and complex microbial communities where *Archaea* are thus theoretically more heavily involved than what will be found using many traditional microcosm/enrichment culture methodologies.

Though this field has made significant advances in the last several years, it is still developing and all forms of research will continue to advance the field. The potential of *Archaea* to serve in bioremediation applications (outside of hypersaline environments) is not well understood. The extremophilic nature of many *Archaea* make them uniquely suitable for biodegradation of “extreme” environments and waste streams, yet many of these possibilities are not yet tested. Future research in bioremediation should be conscious of the potential roles of *Archaea* in bioremediation processes, and thus, methods should be more routinely used to analyze the *Archaea*.

## Figures and Tables

**Table 1:** The strains of hydrocarbon-degrading halophilic *Archaea*.

Strains	Hydrocarbons degraded	Citation
*Haloarcula* st. EH4	Tetradecane, hexadecane, eicosane, heneicosane, pristane, acenaphthene, phenanthrene, anthracene, and 9-methyl anthracene	[14]

*Haloferax* sp. D1227	Benzoate, p-hydroxybenzoate, cinnamate, and phenylpropionate	[20, 21]

*Haloferax mediterranei* st. M-11	Oil	[22]

*Haloarcula* st. D1	4-Hydroxybenzoic acid	[23]

*Haloferax* st. MSNC 4 and MSNC 16 *Haloarcula* sp. st. MSNC 2	Heptadecane	[18]

*Halofera*x st. MSNC 14	Heptadecane, phenanthrene, and pristane	[18, 31]

*Haloferax* sp. HA-1	Crude oil, C8-C34 n-alkanes, benzene, toluene, phenanthrene, biphenyl, and/or naphthalene	[16]
*Haloferax* sp. HA-2		
*Halobacterium* sp. st. HA-3		
*Halococcus* sp. st. HA-4		

*Haloferax alexandrinus* st. B03, B06, AA31, and AA35	Naphthalene, anthracene, phenanthrene, pyrene, and/or benz[a]anthracene	[32]
*Haloferax* sp. SC1-9 st. B07, MM17, AA41, and PR13		
*Haloferax* sp. HSC4 st. MM27		
*Haloferax sulfurifontix* st. CL47		

*Haloferax volcanii* st. DS2	Anthracene	[32]

*Haloterrigena ma*hii sp. H13	Putatively: 1,2-dichloroethane, naphthalene/anthracene, *γ*-hexachlorocyclohexane, 1-/2-methylnapthalene, and benzoate	[35]

*Halobacteriaceae* st. L1	Benzoic and p-hydroxybenzoic acid	[36]

*Natrialba* sp. st. C21	Phenol, naphthalene, and pyrene	[37]

*Haloferax* sp. C-24 and C-27, *Halobacterium piscisalsi* st. C-37, *Halobacterium salinarum* st. C-51, *Halorubrum ezzemoulense* st. C-41 and C-46, *Halorubrum* sp. st. C-43, and *Halobacteriaceae* st. C-50 and C-52	Naphthalene, phenanthrene, pyrene, and/or p-hydroxybenzoate	[39]

*Haloferax lucentense* st. A01	Crude oil, Tween 80, n-octadecane, and phenanthrene	[49, 50]
*Halobacterium salinarum* st. A02		
*Halobacterium* piscisalsi st. A03		
*Haloferax mucosum* st. A04		
*Halobacterium sulfurifontis* st. A05		

*Haloferax elongans* st. M4	Crude oil, n-hexadecane, and phenanthrene as part of a biofilm	[52]
*Halobacterium salinarum* st. M5		

*Halobacterium noricense* st. SA1	Oil, alkanes (C9-C40), benzene, biphenyl, anthracene, naphthalene, and/or phenanthrene	[54]
*Haloferax larsenii* st. SA2, WA3		
*Haloferax elongans* st. SA3, WA1		
*Halobacterium* sp. st. SA4		
*Halobacterium noricense* st. WA2		
*Halobacterium salinarum* st. WA4		

*Haloferax elongans* st. SA3	Crude oil	[55]
*Halobacterium salinarum* st. YS06_13_22		

**Table 2:** The methanogens present in chloroethene-dechlorinating cultures.

Methanogenic strains	Culture notes	Citation
*Methanosarcina* st. KB-1, *Methanomethylovorans* st. KB-1 1 and st. KB-1 2, *Methanomicrobiales* st. KB-1 2, and *Methanosaeta* st. KB-1 1 and st. KB-1 2	*Dehalococcoides*-dominated KB-1 enrichment culture	[155]
Uncultured *Methanobacterium congolense*	*Dehalococcoides*-, *Desulfovibrio*-, and *Clostridia*-dominated ANAS enrichment culture	[152]
*Methanothrix* st. TDC-AR3, *Archaea* st. TDC-AR4, *Methanomethylovorans* st. TDC-AR5, and *Methanothrix* sp. st. TDC-AR6	*Dehalococcoides*- and *Acetobacterium*- containing culture	[153]
Uncultured *Methanosaeta* st. TANA2, uncultured *Methanospirillum* st. TANA5, uncultured *Methanosarcina* st. TANA1, and uncultured *Methanomicrobiales* st. TANA6	Trichloroethene-contaminated aquifer undergoing bioremediation to ethene with a diverse bacterial community	[154]
